# Modern Personalized Strategies for Breast Cancer Treatment: Bridging Precision Oncology and Psycho-Oncology

**DOI:** 10.3390/jcm15041574

**Published:** 2026-02-17

**Authors:** Giuseppe Marano, Ida Paris, Gianandrea Traversi, Osvaldo Mazza, Francesco Pavese, Tatiana D’Angelo, Gianluca Franceschini, Marianna Mazza

**Affiliations:** 1 Department of Neuroscience, Head-Neck and Chest, Section of Psychiatry, Fondazione Policlinico Universitario Agostino Gemelli IRCCS, Largo Agostino Gemelli 8, 00168 Rome, Italy; 2 Department of Neuroscience, Section of Psychiatry, Università Cattolica del Sacro Cuore, 00168 Rome, Italy; 3 Division of Gynecologic Oncology, Department of Woman and Child Health and Public Health, Fondazione Policlinico Universitario A. Gemelli IRCCS, 00168 Rome, Italy; 4 Unit of Medical Genetics, Department of Laboratory Medicine, Ospedale Isola Tiberina-Gemelli Isola, 00186 Rome, Italy; gianandrea.traversi@gmail.com; 5 Spine Surgery Department, Bambino Gesù Children’s Hospital IRCCS, 00168 Rome, Italy; osvaldo.mazza1973@hotmail.it; 6 Breast Surgery Unit, Department of Woman and Child’s Health and Public Health Sciences, Fondazione Policlinico Universitario A. Gemelli IRCCS, 00168 Rome, Italy; 7 Department of Medical and Surgical Sciences, Catholic University of the Sacred Heart, 00168 Rome, Italy

**Keywords:** breast cancer, molecular profiling, targeted therapies, psycho-oncology, psychiatric comorbidity, personalized treatment

## Abstract

Breast cancer represents a paradigmatic model of precision oncology, with treatment strategies increasingly guided by molecular profiling and biomarker-driven targeted therapies. Despite these advances in biological personalization, clinical outcomes remain strongly influenced by psychological and psychiatric factors that are still insufficiently integrated into oncological decision-making. This gap underscores the need for a broader, person-centered model of personalization that extends beyond tumor biology. This narrative review synthesizes current evidence on contemporary personalized strategies in breast cancer management, with a specific focus on the integration of precision oncology and psycho-oncology. A structured literature search was conducted across major biomedical databases to identify studies addressing molecular stratification, targeted treatments, psychiatric comorbidity, psychological profiles, and psycho-oncological interventions relevant to treatment personalization. While molecular classification and biomarker-guided therapies have substantially improved breast cancer outcomes, high rates of depression, anxiety, psychological distress, and maladaptive coping styles are consistently reported across disease stages. These psychological and psychiatric dimensions significantly influence treatment adherence, tolerability, quality of life, and ultimately clinical outcomes. Growing evidence supports the systematic use of psychological screening tools and tailored psycho-oncological interventions, both psychological and pharmacological, as integral components of personalized cancer care. Integrated care models combining oncological and psycho-oncological expertise are associated with improved patient-reported outcomes and may enhance overall therapeutic effectiveness. True personalization in breast cancer treatment extends beyond biological precision and requires the structured integration of psycho-oncological assessment and intervention into routine clinical pathways. Bridging precision oncology and psycho-oncology enables a more comprehensive, patient-centered approach, optimizing adherence, quality of life, and long-term outcomes. Future strategies should prioritize multidisciplinary models of care and the development of integrated clinical frameworks to achieve genuinely personalized breast cancer management.

## 1. Introduction

Breast cancer represents one of the most advanced paradigms of personalized medicine in contemporary oncology. Over the past two decades, the progressive shift from a purely clinicopathological classification toward a molecularly driven framework has profoundly transformed breast cancer diagnosis, prognosis, and treatment selection [1,2], allowing therapeutic decisions to be increasingly tailored to tumor biology rather than solely to anatomical and histological features. The identification of distinct biological subtypes and actionable biomarkers has enabled the development of targeted therapies, leading to significant improvements in survival and disease control across multiple disease stages [3,4], including both early and metastatic settings.

Precision oncology in breast cancer is now firmly grounded in molecular profiling, encompassing hormone receptor status, HER2 expression, genomic signatures, and, more recently, immune and DNA repair-related biomarkers [5,6], which collectively define biologically distinct disease entities and guide treatment selection. These advances have allowed increasingly refined therapeutic stratification, optimizing efficacy while reducing unnecessary toxicity. Nevertheless, despite substantial progress in biological personalization, variability in treatment adherence, tolerance, quality of life, and overall outcomes persists, suggesting that molecular precision alone is insufficient to fully explain clinical heterogeneity [7,8], particularly in real-world clinical practice.

Concurrently, a robust body of evidence indicates that breast cancer is frequently accompanied by significant psychological distress and psychiatric comorbidity. High prevalence rates of depression, anxiety, adjustment disorders, and cancer-related distress have been consistently reported across the disease trajectory, from diagnosis through survivorship and advanced stages [9,10,11]. These psychological dimensions are not merely epiphenomena of the disease experience but exert a measurable impact on treatment-related behaviors, symptom perception, and patient-reported outcomes [12,13,14], thereby influencing both subjective well-being and objective clinical endpoints.

Importantly, psychological and psychiatric factors have been shown to significantly influence adherence to long-term systemic therapies, particularly oral endocrine treatments and targeted agents, which constitute the backbone of modern breast cancer management [8,15]. Depression, anxiety, maladaptive coping styles, and low perceived social support are associated with reduced treatment persistence, increased discontinuation rates, and poorer quality of life, ultimately undermining the potential benefits of precision oncology [16,17], a relationship that underscores the need for integrated biological and psychological assessment. Despite this evidence, psycho-oncological assessment and intervention remain inconsistently implemented in routine oncological care and are often considered ancillary rather than integral to personalized treatment strategies [18,19], resulting in a gap between evidence and clinical practice. Although psychological distress and psychiatric comorbidity are common across multiple cancer populations, breast cancer represents a particularly relevant model for integrated personalization. Its high incidence, prolonged survival trajectories, and frequent reliance on long-term systemic and oral therapies amplify the clinical impact of psychological factors on adherence, tolerability, and quality of life. Moreover, breast cancer care is characterized by complex decision-making processes and extended survivorship phases, during which psychosocial vulnerability may substantially influence real-world treatment effectiveness.

**Figure 1:**
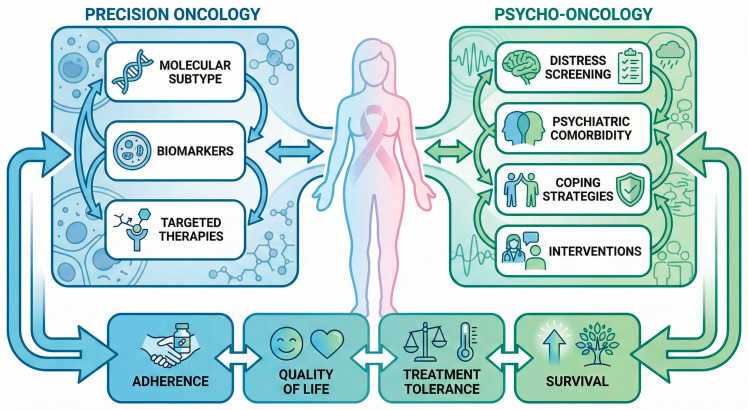
Integration of precision oncology and psycho-oncology in breast cancer care.

Psycho-oncology, as an interdisciplinary field addressing the psychological, psychiatric, social, and behavioral dimensions of cancer, offers validated tools for screening, stratification, and intervention that are directly relevant to treatment personalization [20,21]. Systematic distress screening, psychological profiling, and tailored psychological and pharmacological interventions have demonstrated efficacy in improving emotional well-being, treatment adherence, and patient-reported outcomes in breast cancer populations [22,23]. Emerging integrated care models further suggest that close collaboration between oncologists, psychologists, and psychiatrists may enhance both patient experience and therapeutic effectiveness [24,25], as conceptually integrated within a multidimensional personalization framework (Figure 1).

The figure presents a multidimensional personalized care model combining biological and psycho-oncological determinants of treatment outcomes.

In this context, the concept of personalization in breast cancer requires a conceptual expansion beyond tumor biology alone. True personalized care should encompass both molecular characteristics of the disease and individual psychological vulnerability, resilience, and coping resources. Bridging precision oncology and psycho-oncology represents a critical step toward a more comprehensive, person-centered model of breast cancer treatment, capable of optimizing adherence, quality of life, and long-term clinical outcomes, while addressing the heterogeneity of patient needs beyond biological risk.

Despite substantial advances in biologically driven precision oncology, current personalized treatment frameworks remain largely centered on tumor-related variables, with limited systematic integration of psychological and psychiatric determinants. As a result, personalization is often confined to molecular stratification, while patient-related psychosocial factors that critically influence adherence, tolerability, and real-world effectiveness are addressed inconsistently or reactively. This gap between biological personalization and comprehensive, person-centered care represents a key unmet need in contemporary breast cancer management.

The aim of this narrative review is to synthesize current evidence on modern personalized strategies in breast cancer treatment, with a specific focus on the integration of precision oncology and psycho-oncology. By examining biological stratification, psychological and psychiatric dimensions, and emerging integrated care models, this review seeks to highlight the clinical relevance of a multidimensional approach to personalization and to outline future directions for truly integrated breast cancer management. In this review, the term “modern personalized strategies” refers not only to recent molecularly driven advances in precision oncology, but also to the broader conceptual shift toward multidimensional personalization, integrating biological, psychological, and psychiatric determinants of treatment outcomes.

## 2. Materials and Methods

### 2.1. Literature Search Strategy

A structured literature search was conducted to identify relevant studies examining personalized treatment strategies in breast cancer, with particular emphasis on the integration of precision oncology and psycho-oncology. The search was performed across the following electronic databases: PubMed/MEDLINE, Scopus, Web of Science, and PsycINFO. Articles published in English from January 2000 to December 2024 were considered eligible, reflecting the emergence and evolution of molecular classification in breast cancer and the parallel development of psycho-oncology as a clinical discipline, thereby capturing both early molecular advances and the integration of psychosocial dimensions over time.

The search strategy combined Medical Subject Headings (MeSH) terms and free-text keywords related to breast cancer, precision oncology, molecular profiling, targeted therapies, psycho-oncology, psychiatric comorbidity, psychological distress, quality of life, and treatment adherence. Boolean operators (“AND”, “OR”) were used to refine the search. Reference lists of relevant articles and previous reviews were also manually screened to identify additional pertinent studies, in order to minimize publication bias and include seminal or overlooked works.

### 2.2. Eligibility Criteria

Studies were included if they met the following criteria: involved adult patients diagnosed with breast cancer at any disease stage; addressed molecular stratification, biomarker-driven therapies, or precision oncology approaches and/or psychological, psychiatric, or psycho-oncological dimensions relevant to treatment personalization; consisted of original clinical studies (randomized controlled trials, cohort studies, case–control studies), systematic reviews, or meta-analyses; published in peer-reviewed journals.

Exclusion criteria comprised case reports, small case series, conference abstracts, editorials, letters to the editor, and studies lacking clinical relevance to personalized treatment strategies. Preclinical studies were excluded unless they provided direct translational implications for clinical practice, thus maintaining the clinical applicability of the evidence synthesis.

### 2.3. Study Selection and Data Extraction

Two authors (G.M. and M.M) independently screened titles and abstracts for eligibility. Full texts of potentially relevant articles were retrieved and assessed for inclusion. Discrepancies in study selection were resolved through discussion and consensus, ensuring methodological rigor and minimizing subjective bias. From each included study, data were extracted regarding study design, population characteristics, breast cancer subtype, type of personalized oncological strategy, psychological or psychiatric variables assessed, psycho-oncological interventions, and reported clinical or patient-reported outcomes, with particular attention to variables influencing adherence, quality of life, and survival outcomes.

### 2.4. Narrative Synthesis

Given the heterogeneity of study designs, populations, outcome measures, and intervention types, a quantitative meta-analysis was not feasible. Therefore, findings were synthesized using a narrative and thematic approach. Studies were grouped into key domains, including molecular classification and targeted therapies, prevalence and impact of psychological and psychiatric comorbidities, effects of psychological factors on clinical outcomes, psycho-oncological interventions, and integrated models of care, allowing a multidimensional perspective on personalized breast cancer management.

**Figure 2:**
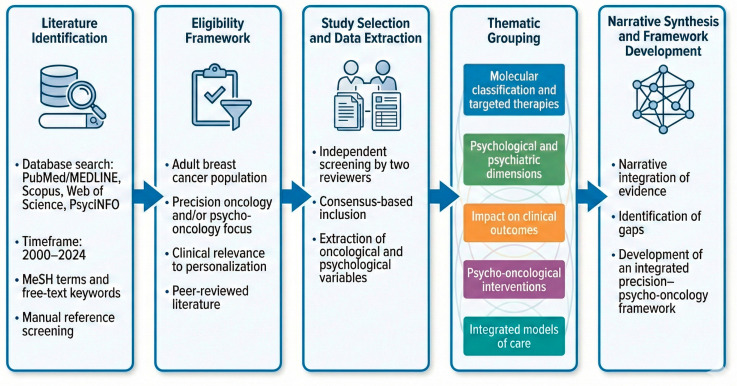
Conceptual flow of literature selection and narrative synthesis.

This narrative synthesis aimed to highlight converging evidence across biological and psychosocial domains, identify gaps in current personalized treatment paradigms, and propose an integrated framework bridging precision oncology and psycho-oncology in breast cancer management, providing a roadmap for truly person-centered therapeutic strategies. The overall process of literature identification, thematic grouping, and narrative integration is schematically illustrated in Figure 2.

This figure illustrates the structured process used to identify, select, and narratively synthesize the literature on personalized breast cancer care. The diagram depicts the sequential phases of literature identification, eligibility framing, study selection, thematic grouping, and narrative integration across precision oncology and psycho-oncology domains. The figure highlights the conceptual logic underlying evidence integration and framework development in this narrative review.

Psychological and psychiatric dimensions were included if assessed using validated diagnostic criteria or standardized screening instruments. Given heterogeneity in assessment methods across studies, findings were interpreted thematically rather than quantitatively. To mitigate selection bias inherent to narrative reviews, the literature search followed predefined inclusion and exclusion criteria, was conducted across multiple databases, and involved independent screening by two authors. Disagreements were resolved by consensus. In addition, reference lists of relevant articles were manually screened to identify seminal or overlooked studies, aiming to ensure balanced and comprehensive coverage of the literature.

## 3. Precision Oncology in Breast Cancer

Precision oncology has fundamentally reshaped breast cancer management by enabling treatment strategies tailored to the biological characteristics of individual tumors. The transition from traditional histopathological classification to molecularly informed frameworks has allowed more accurate prognostic stratification and the rational selection of targeted therapies, significantly improving clinical outcomes across disease stages [1,2,3], and providing the biological backbone for integration with psycho-oncological interventions.

### 3.1. Molecular Classification of Breast Cancer

Molecular classification represents the cornerstone of personalized breast cancer treatment. Gene expression profiling has identified biologically distinct subtypes—luminal A, luminal B, HER2-enriched, and basal-like, which broadly correspond to clinically relevant categories based on hormone receptor (HR) and HER2 status [1,2]. In routine clinical practice, surrogate immunohistochemical markers, including estrogen receptor (ER), progesterone receptor (PR), HER2 expression, and Ki-67 proliferation index, are commonly used to guide therapeutic decisions [26], enabling rapid, cost-effective stratification when genomic assays are not available.

Hormone receptor-positive/HER2-negative breast cancer constitutes the most prevalent subtype and is characterized by a generally favorable prognosis and sensitivity to endocrine therapies. HER2-positive breast cancer, once associated with aggressive clinical behavior, has become a paradigmatic example of successful targeted therapy development. Triple-negative breast cancer (TNBC), defined by the absence of ER, PR, and HER2 expression, remains biologically heterogeneous and clinically challenging, with limited targeted treatment options and poorer overall outcomes [27,28], highlighting the ongoing need for novel biomarkers and therapeutic strategies.

Beyond intrinsic subtypes, multigene assays such as Oncotype DX, MammaPrint, and PAM50 have further refined risk stratification and treatment personalization, particularly in early-stage HR-positive disease, by informing decisions regarding the addition of chemotherapy to endocrine therapy [29,30]. These tools exemplify the increasing granularity of biological personalization in breast cancer care.

### 3.2. Biomarker-Driven and Targeted Therapies

The integration of molecular biomarkers into clinical decision-making has led to the development of highly effective targeted therapies. Beyond merely identifying tumor subtypes, biomarkers now guide therapy selection, predict resistance mechanisms, and allow dynamic treatment adaptation based on longitudinal monitoring of tumor evolution. Endocrine therapy remains the backbone of treatment for HR-positive disease, with aromatase inhibitors, selective estrogen receptor modulators, and selective estrogen receptor degraders demonstrating substantial benefits in both early and advanced settings [31]. Recent research emphasizes the role of genomic signatures (e.g., ESR1 mutations) in predicting endocrine resistance, enabling personalized escalation or de-escalation strategies.

The advent of CDK4/6 inhibitors has further transformed advanced HR-positive breast cancer management, not only improving progression-free and overall survival, but also influencing quality-of-life outcomes by maintaining disease control with manageable toxicity [32,33]. Similarly, HER2-targeted therapies, including monoclonal antibodies and antibody–drug conjugates, have dramatically improved outcomes for patients with HER2-positive disease, extending survival even in metastatic settings [34,35].

In TNBC and selected HR-positive tumors, therapeutic exploitation of DNA damage repair defects through PARP inhibitors has introduced precision oncology approaches based on germline and somatic BRCA mutations. This highlights the paradigm shift from static classification to functional biomarker-driven therapy [36,37]. More recently, immune checkpoint inhibitors have demonstrated efficacy in subsets of TNBC characterized by immune activation, underscoring the growing role of tumor microenvironment profiling in therapeutic personalization [38].

These advances underscore the success of biomarker-driven treatment strategies in improving disease control while emphasizing the increasing complexity of therapeutic decision-making.

### 3.3. Clinical Impact and Limitations of Biological Personalization

Despite the undeniable benefits of precision oncology, several limitations persist. First, biological stratification does not fully account for the wide interindividual variability observed in treatment tolerance, adherence, and patient-reported outcomes. Even within molecularly homogeneous subgroups, patients may experience markedly different trajectories in terms of symptom burden, quality of life, and treatment persistence [3,8].

Second, the growing use of long-term oral therapies, including endocrine agents and targeted treatments, has shifted a substantial portion of treatment responsibility to patients, making adherence a critical determinant of therapeutic success. Non-adherence and early discontinuation remain common and are only partially explained by biological or pharmacological factors [8,15].

Finally, the biological focus of precision oncology often overlooks psychosocial vulnerability, psychiatric comorbidity, and individual coping resources, which can profoundly influence treatment engagement and outcomes. As a result, biologically optimal therapies may fail to deliver their full clinical benefit when psychological and psychiatric dimensions are not adequately addressed.

Collectively, these considerations highlight the need to complement biological precision with a broader, person-centered approach to personalization. While molecular stratification remains indispensable, it represents only one component of truly personalized breast cancer care. Integrating psycho-oncological perspectives alongside precision oncology may help bridge existing gaps between biological efficacy and real-world clinical effectiveness.

Although precision oncology has substantially advanced breast cancer management, biological stratification alone does not fully capture the multidimensional determinants of treatment effectiveness. Table 1 summarizes the complementary roles of precision oncology and psycho-oncology within personalized breast cancer care, highlighting their distinct targets, tools, and clinical outcomes.

### 3.4. Artificial Intelligence, Multi-Omics, and Emerging Technologies in Precision Breast Cancer Oncology

Beyond conventional molecular stratification, emerging technologies such as artificial intelligence (AI), multi-omics data integration, and digital pathology are increasingly reshaping contemporary precision oncology in breast cancer. These approaches aim to manage the growing complexity of biological data and to refine prognostic and therapeutic stratification beyond traditional clinicopathological variables.

AI-based models have demonstrated increasing utility in breast cancer risk assessment, screening, prognostic prediction, and treatment optimization, particularly through the application of deep learning algorithms to imaging, histopathology, and integrated clinical datasets [39,40,41]. Recent AI-driven scoring systems combining clinical, pathological, and molecular variables enable the stratification of breast cancer patients into subgroups with distinct prognostic trajectories and therapeutic needs, supporting more individualized treatment strategies [41]. These approaches illustrate the transition from static biomarker-based classification to dynamic, data-driven models of biological personalization.

Multi-omics integration represents another major advance in precision oncology, allowing the combined analysis of genomic, transcriptomic, epigenomic, and proteomic data to capture tumor heterogeneity and biological complexity at an unprecedented resolution [42,43]. Large-scale genomic studies have refined intrinsic breast cancer subtypes and revealed substantial inter- and intratumoral heterogeneity, including discordance between primary tumors and metastatic lesions, with important implications for treatment selection and resistance mechanisms [44]. These findings highlight the limitations of single-time-point molecular assessment and the need for more dynamic approaches to tumor characterization.

Single-cell transcriptomic analyses have further expanded current understanding of breast cancer biology by uncovering distinct cellular states within tumors and the tumor microenvironment that are not detectable using bulk sequencing techniques [45]. In aggressive subtypes such as triple-negative breast cancer, single-cell approaches have identified biologically and clinically relevant subpopulations associated with immune evasion, therapy resistance, and differential clinical outcomes, supporting the development of more refined, mechanism-based therapeutic strategies [46,47].

In parallel, digital pathology has emerged as a powerful platform for AI-based biomarker discovery and clinical decision support. Computational analysis of whole-slide images enables the extraction of quantitative features related to tumor architecture, immune infiltration, and stromal composition, offering complementary biological information beyond conventional histopathological evaluation [48,49]. These technologies hold promise for scalable and reproducible precision oncology applications in routine clinical practice.

While these technological innovations significantly enhance biological personalization, they also increase the complexity of treatment pathways and patient decision-making. The growing use of AI-driven risk stratification, multi-omics profiling, and advanced diagnostic technologies underscores the need to integrate psycho-oncological assessment alongside biological precision. Patients confronted with complex prognostic information and long-term targeted therapies may experience heightened psychological burden, which can directly influence treatment adherence, tolerability, and real-world effectiveness. Consequently, advances in precision oncology further reinforce the necessity of a parallel, structured psycho-oncological personalization framework to ensure that biologically optimized therapies achieve their full clinical potential.

## 4. Psychological and Psychiatric Dimensions in Breast Cancer

Psychological and psychiatric dimensions represent a highly prevalent and clinically relevant component of the breast cancer experience, with significant implications for treatment personalization. These dimensions not only reflect the emotional impact of the disease but actively modulate treatment adherence, symptom perception, and overall patient outcomes, highlighting their integration as a core element of personalized care. Across disease stages, patients frequently exhibit a spectrum of emotional and psychiatric symptoms that interact with oncological treatments and influence both patient-reported and clinical outcomes. The interindividual variability in psychological response underscores the need for tailored assessment and intervention within a personalized care framework.

### 4.1. Depression in Breast Cancer

Depression is one of the most extensively studied psychiatric conditions in breast cancer, with prevalence estimates ranging from 10% to 30%, depending on disease stage, assessment method, and timing of evaluation [9,10]. Both major depressive disorder and subthreshold depressive symptoms are commonly observed, particularly during active treatment phases and in patients receiving long-term systemic therapies [11].

Clinically, depression in breast cancer is associated with increased symptom burden, reduced quality of life, impaired functional status, and greater healthcare utilization [12]. Depression can also dysregulate hypothalamic–pituitary–adrenal axis function and inflammatory pathways, potentially influencing tumor biology and recovery, further underlining the clinical relevance of its assessment. From a treatment perspective, depressive symptoms have been consistently linked to poorer adherence to endocrine therapy and other oral anticancer agents, leading to reduced treatment persistence and premature discontinuation [8,15]. Depression may also amplify the perception of treatment-related side effects, such as fatigue, pain, and cognitive complaints, further compromising tolerability and engagement with care.

Beyond its impact on patient-reported outcomes, depression has been associated with adverse oncological outcomes in some studies, including reduced survival, although causality remains complex and likely mediated by behavioral, biological, and social factors [50]. These findings highlight depression as a critical, clinically actionable dimension within personalized breast cancer management.

### 4.2. Anxiety Disorders and Anxiety Symptoms

Anxiety is highly prevalent in breast cancer patients, particularly around diagnosis, treatment initiation, and disease surveillance. Reported prevalence rates of clinically significant anxiety symptoms range from 20% to 40%, with both generalized anxiety and cancer-specific fears contributing to overall psychological burden [51,52].

From a clinical standpoint, anxiety is closely linked to heightened symptom vigilance, anticipatory distress, and increased reporting of treatment-related adverse effects. Severe anxiety may also interfere with decision-making capacity, resulting in delayed consent for complex treatments or reluctance to participate in clinical trials. While moderate anxiety may facilitate engagement with care, excessive or persistent anxiety can interfere with decision-making, impair communication with healthcare providers, and negatively affect adherence to complex treatment regimens [17].

Anxiety has also been associated with reduced quality of life and increased utilization of supportive care resources. In the context of precision oncology, where treatment choices may involve nuanced risk–benefit discussions and long-term oral therapies, unmanaged anxiety may limit the effective implementation of biologically optimal treatment strategies.

### 4.3. Cancer-Related Distress as a Transversal Construct

Cancer-related distress represents a broader, transdiagnostic construct encompassing emotional, cognitive, social, and existential responses to cancer and its treatment. Unlike formal psychiatric diagnoses, distress captures the dynamic psychological burden experienced by patients and is particularly useful for clinical screening and stratification [20].

High levels of distress are consistently reported in breast cancer populations and may fluctuate over time in response to disease milestones, treatment transitions, and survivorship challenges [21]. Clinically relevant distress has been associated with poorer treatment adherence, reduced satisfaction with care, and impaired quality of life [23]. Importantly, distress often coexists with subthreshold depressive and anxiety symptoms that may not meet diagnostic criteria but still exert a meaningful impact on outcomes.

The routine assessment of distress using validated screening tools has been recommended by international guidelines as a standard component of comprehensive cancer care [20]. Within a personalized treatment paradigm, distress screening allows early identification of vulnerable patients and facilitates targeted psycho-oncological interventions.

### 4.4. Psychological Profiles, Coping Styles, and Clinical Relevance

Beyond discrete symptom domains, individual psychological profiles and coping styles play a crucial role in shaping patients’ responses to breast cancer and its treatment. Maladaptive coping strategies, such as avoidance, denial, catastrophizing, and emotional suppression, correlate with elevated distress, decreased adherence, and poorer quality of life [12,16]. Adaptive coping strategies, resilience, problem-solving skills, and social support, conversely, enhance treatment persistence and overall well-being.

From a clinical perspective, these psychological characteristics contribute to heterogeneity in treatment tolerance and adherence that cannot be explained by tumor biology alone. As breast cancer management increasingly relies on long-term, patient-administered therapies, understanding psychological vulnerability and coping resources becomes essential for effective personalization of care.

## 5. Impact of Psychological Factors on Clinical Outcomes

Psychological and psychiatric factors exert a substantial and clinically measurable impact on breast cancer outcomes, influencing not only patient-reported measures but also key treatment-related behaviors and, indirectly, disease prognosis. As breast cancer management increasingly relies on long-term systemic and oral therapies, understanding the role of psychological determinants has become essential to translating biological efficacy into real-world effectiveness. Proposed mechanisms linking psychological factors to clinical outcomes include behavioral pathways (reduced adherence, delayed care-seeking), biological pathways (neuroendocrine and inflammatory dysregulation), and healthcare-related factors (impaired communication and engagement). These mechanisms may vary according to disease stage, treatment modality, and molecular subtype, although current evidence remains predominantly observational.

### 5.1. Treatment Adherence and Persistence

Adherence to prescribed anticancer therapies represents a critical determinant of clinical outcomes in breast cancer. Non-adherence and early discontinuation are particularly prevalent in the context of long-term oral treatments, such as adjuvant endocrine therapy, where adherence rates decline progressively over time [15]. Psychological factors, especially depression and anxiety, have been consistently identified as major contributors to suboptimal adherence.

Depressive symptoms are strongly associated with reduced treatment persistence, increased rates of missed doses, and premature discontinuation of endocrine therapy, ultimately compromising its therapeutic benefit [13,15]. Anxiety and distress further exacerbate adherence challenges by amplifying concerns about side effects and diminishing patients’ confidence in long-term treatment necessity. Importantly, these associations persist after adjustment for clinical and demographic variables, underscoring the independent contribution of psychological factors to treatment adherence.

### 5.2. Treatment Tolerability and Symptom Burden

Psychological vulnerability significantly influences the perception, reporting, and tolerability of treatment-related side effects. Patients with elevated levels of depression, anxiety, or distress frequently report greater symptom burden, including fatigue, pain, cognitive complaints, and vasomotor symptoms, even when receiving identical treatment regimens [12,17].

This heightened symptom perception may lead to dose reductions, treatment interruptions, or discontinuation, thereby limiting therapeutic efficacy. The nocebo effect, where negative expectations exacerbate perceived adverse effects, has been increasingly recognized as a clinically relevant phenomenon in oncology, particularly among psychologically distressed patients [53]. In the context of precision oncology, where therapies are selected based on biological appropriateness, psychological determinants of tolerability represent a critical, yet often overlooked, modifier of treatment success.

### 5.3. Quality of Life and Functional Outcomes

Quality of life (QoL) is a central outcome in breast cancer care, particularly given improved survival rates and the chronic nature of many treatments. Psychological distress, depression, and anxiety are among the strongest predictors of impaired QoL across physical, emotional, and social domains [11,54]. Poor QoL is, in turn, associated with reduced treatment engagement, lower adherence, and diminished satisfaction with care.

From a clinical standpoint, impaired QoL may signal unmet supportive care needs and identify patients at risk for suboptimal treatment outcomes. Patient-reported outcome measures increasingly incorporated into clinical trials and real-world studies provide valuable insights into the interplay between psychological factors and therapeutic effectiveness, reinforcing the relevance of psychosocial assessment within personalized treatment strategies.

### 5.4. Psychological Factors and Survival Outcomes

The relationship between psychological factors and survival outcomes in breast cancer remains complex and partially mediated by behavioral and biological pathways. Several observational studies and meta-analyses have reported associations between depression and increased mortality in cancer populations, including breast cancer [50]. Proposed mechanisms include reduced treatment adherence, unhealthy health behaviors, dysregulation of neuroendocrine and immune pathways, and delayed healthcare utilization.

While causality cannot be definitively established, the consistent association between psychological morbidity and adverse outcomes supports the clinical relevance of psychological assessment and intervention. Importantly, even in the absence of direct effects on survival, the impact of psychological factors on adherence, tolerability, and quality of life provides a strong rationale for their integration into outcome-oriented personalized care models.

## 6. Psycho-Oncological Interventions as Personalized Strategies: A Clinical Toolbox

**Figure 3:**
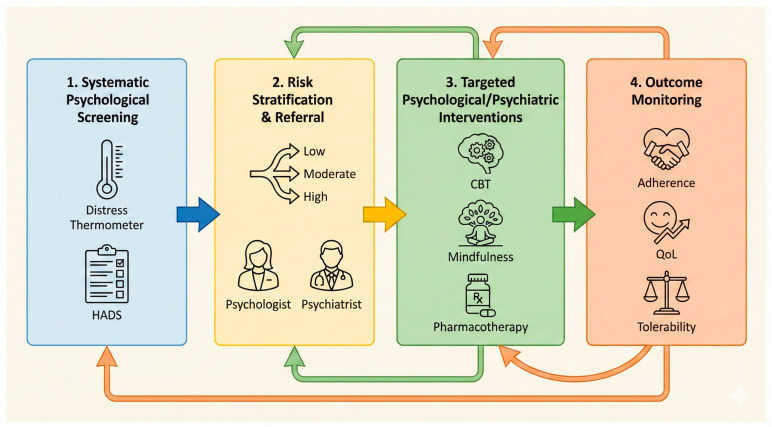
Conceptual Framework for Integrating Psycho-Oncology into Personalized Breast Cancer Care.

The integration of psycho-oncological care into personalized breast cancer management requires a structured and clinically actionable framework. Rather than being viewed as an adjunctive or optional component, psycho-oncology can be conceptualized as a toolbox that enables systematic identification of psychological vulnerability, targeted intervention, and measurable improvements in treatment-related outcomes. This approach aligns with the principles of precision medicine by tailoring supportive care to individual patient needs (Figure 3).

The above figure contains a conceptual diagram illustrating a four-step, personalized psycho-oncology approach integrated into breast cancer care: (1) systematic psychological screening; (2) risk stratification and personalized referral; (3) targeted psychological and psychiatric interventions; and (4) longitudinal outcome monitoring.

### 6.1. Step 1: Systematic Psychological Screening

Routine psychological screening represents the entry point for personalized psycho-oncological care. Validated screening tools allow rapid identification of patients experiencing clinically relevant levels of distress, depression, or anxiety, including those who may not spontaneously report psychological symptoms.

Commonly used instruments in breast cancer care include the Distress Thermometer, the Hospital Anxiety and Depression Scale (HADS), and brief patient-reported outcome measures assessing emotional well-being [9,19,55]. These tools are feasible in routine clinical settings and have demonstrated sensitivity in detecting patients at risk for poor adherence, reduced quality of life, and impaired treatment tolerance.

From a clinical perspective, systematic screening enables early identification of vulnerable patients at key time points, such as diagnosis, treatment initiation, transitions between treatment phases, and survivorship. Importantly, screening should not be a one-time assessment but rather a dynamic process reflecting the evolving psychological burden across the disease trajectory. Commonly adopted cutoff values, such as a Distress Thermometer score ≥4 or HADS subscale scores ≥8, are frequently used to identify clinically relevant psychological distress warranting further evaluation. Reassessment is recommended at key clinical milestones, including diagnosis, treatment initiation, transitions between treatment phases, and follow-up visits. While initial screening may be performed by oncology staff, interpretation of results and intervention planning should involve psychologists or psychiatrists within the multidisciplinary team.

### 6.2. Step 2: Risk Stratification and Personalized Referral

Following screening, patients can be stratified according to the severity and nature of psychological symptoms, allowing personalized referral pathways. Patients with mild or transient distress may benefit from structured psychoeducational interventions and supportive counseling, whereas those with moderate to severe symptoms often require specialized psychological or psychiatric care.

Risk stratification should consider not only symptom severity but also individual coping styles, social support, previous psychiatric history, and treatment complexity. In the context of long-term oral therapies, such as endocrine treatment or targeted agents, patients exhibiting depressive symptoms or maladaptive coping patterns represent a high-risk group for non-adherence and early discontinuation [8,16].

This stratified approach optimizes resource allocation and ensures that psycho-oncological interventions are proportionate, timely, and clinically relevant.

### 6.3. Step 3: Targeted Psychological and Psychiatric Interventions

A range of evidence-based psycho-oncological interventions is available and can be tailored to individual patient profiles. Psychological interventions, including cognitive-behavioral therapy, supportive-expressive therapy, and mindfulness-based interventions, have demonstrated efficacy in reducing depression, anxiety, and cancer-related distress in breast cancer patients [22,23].

For patients with clinically significant depressive or anxiety disorders, psychiatric interventions, including pharmacological treatment, may be indicated. Antidepressants and anxiolytics, when appropriately selected and monitored, can improve mood symptoms and functional status, thereby facilitating treatment adherence and engagement with oncological care [24]. Attention to potential drug–drug interactions with anticancer therapies is essential, reinforcing the importance of close collaboration between oncologists and mental health professionals.

Importantly, psycho-oncological interventions should be integrated into the overall treatment plan rather than delivered in isolation. Alignment with oncological milestones and treatment schedules enhances acceptability and clinical impact. For example, a patient initiating long-term adjuvant endocrine therapy who presents with subthreshold depressive symptoms and maladaptive coping may not require immediate psychiatric treatment but can benefit from early psychoeducational interventions and supportive counseling to prevent future non-adherence. Conversely, a patient with clinically significant depression and poor social support may require prompt referral for specialized psychological or psychiatric care to ensure treatment persistence and tolerability.

### 6.4. Step 4: Impact on Clinical and Patient-Reported Outcomes

The implementation of structured psycho-oncological care has been associated with meaningful improvements in clinically relevant outcomes. Evidence indicates that targeted psychological and psychiatric interventions can enhance treatment adherence, reduce premature discontinuation of endocrine and targeted therapies, and improve symptom tolerability [8,17].

In addition to adherence-related benefits, psycho-oncological interventions consistently improve quality of life, emotional functioning, and patient satisfaction with care. While direct effects on survival outcomes remain difficult to establish, the cumulative impact on treatment persistence, tolerability, and functional status supports a clinically meaningful role for psycho-oncology within personalized breast cancer management.

From an outcome-oriented perspective, psycho-oncology functions as a critical enabler of precision oncology, ensuring that biologically optimal treatments can be delivered effectively in real-world clinical settings.

To translate psycho-oncological principles into routine clinical practice, a structured and reproducible framework is required. Table 2 outlines a stepwise integrated precision–psycho-oncology model, illustrating key clinical actions, tools, and expected outcomes that can be implemented across oncology centers.

## 7. Integrated Models of Care: A Replicable Framework for Oncology Centers

The effective integration of precision oncology and psycho-oncology requires organizational models that are both clinically meaningful and operationally feasible. The translation of research evidence into routine clinical practice is often hindered by fragmented services and lack of structured workflows; integrated care models provide a solution by aligning biological and psychosocial management strategies systematically. To translate evidence into routine practice, integrated care models must be structured, scalable, and adaptable to different oncology settings. In this context, psycho-oncology should be embedded within standard breast cancer pathways rather than delivered as an optional or reactive service. Embedding psycho-oncological expertise within existing care pathways enhances timely identification of at-risk patients and facilitates proactive, rather than reactive, interventions.

### 7.1. Core Components of an Integrated Precision–Psycho-Oncology Model

A replicable integrated model for breast cancer care is based on four core components: multidisciplinary collaboration, standardized screening, stratified intervention pathways, and outcome monitoring.

First, multidisciplinary collaboration represents the structural foundation of integration. Oncologists, breast surgeons, radiation oncologists, psychologists, psychiatrists, oncology nurses, and supportive care professionals should operate within a shared clinical framework, with clearly defined roles and communication channels [20]. Psycho-oncological expertise should be available within the breast cancer team and involved in treatment planning, particularly for patients receiving long-term systemic therapies. Close collaboration ensures that patient-specific psychological vulnerabilities are considered alongside tumor biology, which can directly influence treatment adherence and tolerability.

Second, standardized psychological screening should be incorporated into routine clinical workflows. Screening at predefined time points, such as diagnosis, treatment initiation, therapy transitions, and follow-up visits, ensures early identification of psychological vulnerability. The use of brief, validated tools allows screening to be performed efficiently without disrupting clinical throughput [9]. Routine screening enables stratification of patients, allowing early interventions that can prevent escalation of distress and improve adherence to long-term therapies.

Third, stratified intervention pathways enable tailored responses based on patient needs. Low-risk patients may benefit from psychoeducation and supportive counseling, while high-risk patients, such as those with depression, significant anxiety, or adherence difficulties, should be promptly referred for specialized psychological or psychiatric care. This stepped-care approach optimizes resource allocation and enhances clinical effectiveness [21]. This stepped-care approach balances resource utilization with clinical effectiveness, ensuring that patients receive the intensity of care proportional to their psychosocial risk profile.

Finally, systematic outcome monitoring is essential to evaluate the clinical impact of integrated care. Both patient-reported outcomes (e.g., quality of life, distress levels) and clinical indicators (e.g., treatment adherence, persistence, dose modifications) should be routinely assessed to inform ongoing care and service improvement. Monitoring provides data to continuously refine intervention pathways and supports evidence-based adjustments in real-time patient management.

The proposed model does not represent a single alternative pathway but rather a concurrent integration of precision oncology and psycho-oncology. Molecular-driven treatment selection remains central to clinical decision-making, while psycho-oncological assessment operates in parallel as an enabling component that enhances treatment feasibility, adherence, and tolerability. In situations of limited resources, a stepped-care approach prioritizing high-risk patients allows pragmatic reconciliation between biological urgency and psychosocial needs.

### 7.2. Integration into Clinical Pathways and Decision-Making

For integration to be sustainable, psycho-oncological assessment and intervention must be aligned with existing breast cancer clinical pathways. Psychological screening results should be documented in the medical record and considered during multidisciplinary tumor board discussions when relevant. Incorporating psychosocial data into clinical decision-making ensures that treatment plans are feasible for the patient and maximizes the likelihood of adherence. This ensures that treatment decisions account not only for tumor biology but also for patient-specific psychological factors that may influence treatment feasibility and adherence.

In particular, patients initiating long-term oral therapies, such as adjuvant endocrine treatment or targeted agents, represent a priority group for integrated care. Early identification of psychological vulnerability in these patients allows proactive intervention, reducing the risk of non-adherence and premature discontinuation [8,56]. Targeting these high-risk groups can significantly improve long-term clinical outcomes by preventing treatment interruptions that compromise the effectiveness of precision therapies. Screening tools typically require less than five minutes and can be administered by oncology nurses or clinical staff. Interpretation and intervention planning should involve psychologists or psychiatrists integrated within the multidisciplinary team. Embedding psycho-oncological data into tumor board discussions ensures feasibility without disrupting workflow.

### 7.3. Feasibility and Scalability Across Oncology Settings

A key advantage of integrated precision–psycho-oncology models is their scalability. While comprehensive psycho-oncology services may not be immediately available in all centers, core elements, such as routine screening and structured referral pathways, can be implemented incrementally. Telemedicine platforms, digital monitoring, and remote psychological consultations allow smaller or resource-limited centers to provide timely interventions without requiring full on-site psycho-oncology teams.

Importantly, integration does not necessarily require extensive additional resources but rather a reorganization of existing services and workflows. Training oncology staff to recognize psychological distress and establishing clear referral criteria allows efficient utilization of available resources while maintaining high standards of patient-centered care.

### 7.4. Clinical and Organizational Benefits

Integrated care models have demonstrated benefits at both the patient and system levels. Clinically, they are associated with improved emotional well-being, enhanced treatment adherence, better symptom management, and higher patient satisfaction [22,23]. Psychosocially supported patients are more likely to complete their prescribed treatment courses, leading to higher real-world effectiveness of precision oncology interventions. From an organizational perspective, improved adherence and reduced treatment discontinuation may translate into more efficient use of oncological therapies and supportive care resources.

Moreover, integrated models support a shift toward truly personalized medicine, in which treatment selection and delivery are informed by both biological and psychosocial determinants [57]. This alignment ensures that the precision of molecularly targeted therapies is matched by individualized support for psychological resilience and coping, creating a more holistic treatment paradigm. Beyond clinical and patient-reported outcomes, integrated precision–psycho-oncology models may also influence healthcare utilization and cost-effectiveness by reducing unplanned visits, treatment discontinuation, and inefficient use of oncological therapies. Improved patient satisfaction and engagement represent additional relevant outcomes, associating integrated care with emerging value-based oncology frameworks. Current evidence supporting integrated oncology–psycho-oncology models derives primarily from observational studies and interventional trials focusing on patient-reported outcomes. Robust randomized evidence for survival benefits remains limited. Nevertheless, consistent improvements in adherence, quality of life, and patient satisfaction provide clinically meaningful justification for implementation.

### 7.5. Toward Standardization of Integrated Personalized Care

Despite growing evidence, the implementation of integrated precision–psycho-oncology models remains heterogeneous across oncology centers. Standardizing key components, such as screening protocols, referral pathways, and outcome metrics, could facilitate broader adoption and benchmarking. Consensus-driven guidelines and quality indicators would allow centers to compare performance, optimize workflow, and ensure equitable access to psycho-oncological services. The development of consensus-based guidelines and quality indicators may further support integration into routine breast cancer care.

In summary, integrated models bridging precision oncology and psycho-oncology offer a replicable and scalable framework for personalized breast cancer management. By embedding psycho-oncological assessment and intervention into standard clinical pathways, oncology centers can enhance the real-world effectiveness of precision therapies and move toward a more comprehensive, person-centered model of care.

**Figure 4:**
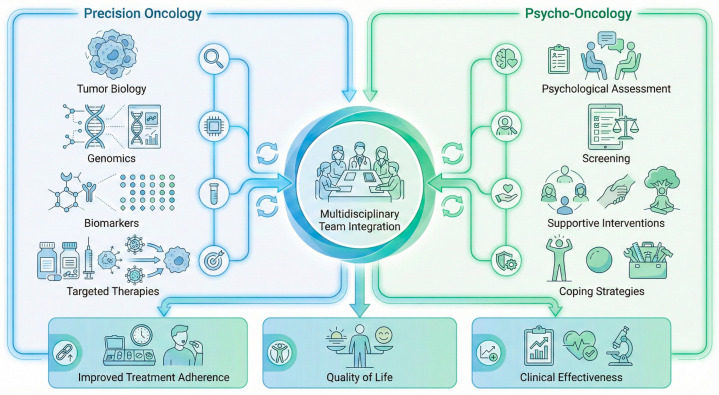
Integrated Precision–Psycho-Oncology Framework for Personalized Breast Cancer Care.

The integrated precision–psycho-oncology framework described above is schematically illustrated in Figure 4, highlighting the interaction between tumor biological characteristics, patient-related psychological factors, and multidisciplinary care pathways leading to improved clinical outcomes.

This figure illustrates an integrated framework in which tumor biological characteristics and patient-related psychological and psychiatric factors jointly inform personalized breast cancer management. Precision oncology enables biologically driven treatment selection, while psycho-oncological assessment identifies psychological vulnerability and guides targeted supportive interventions. Their integration within multidisciplinary care pathways aims to optimize treatment adherence, tolerability, quality of life, and overall clinical effectiveness.

## 8. Toward Truly Personalized Breast Cancer Care

The concept of personalization in breast cancer care is undergoing a necessary evolution. While precision oncology has successfully refined treatment selection based on tumor biology, emerging evidence underscores that biological stratification alone is insufficient to ensure optimal clinical outcomes. True personalization requires acknowledging that patient outcomes are shaped not only by molecular targets but also by psychosocial, behavioral, and psychiatric factors that interact dynamically with treatment regimens [57,58]. Truly personalized breast cancer care requires the systematic integration of psychological, psychiatric, and behavioral dimensions into standard oncological pathways, thereby aligning therapeutic efficacy with real-world effectiveness.

A key priority for advancing personalized care lies in the routine incorporation of psycho-oncological assessment into clinical decision-making. Standardized psychological screening at critical time points, such as diagnosis, treatment initiation, and transitions to long-term systemic therapies, should become an integral component of breast cancer management rather than an optional adjunct [20]. Systematic screening allows clinicians to anticipate barriers to adherence, identify emerging distress, and implement timely interventions that prevent deterioration in both psychological and clinical outcomes. Such an approach enables early identification of patients at risk for poor adherence, reduced tolerability, and impaired quality of life, allowing timely and targeted intervention.

Future personalized strategies should also move beyond symptom-based assessment toward multidimensional risk profiling. Integrating psychological variables, such as depressive symptoms, anxiety levels, coping styles, and social support, alongside biological and clinical factors may enhance the precision of treatment planning. This multidomain stratification model recognizes the complex interplay between tumor biology, patient vulnerability, and therapy demands, which is especially relevant for long-term oral or targeted therapies where adherence and tolerability critically influence effectiveness [8,12].

Digital health technologies and remote monitoring tools offer additional opportunities to support integrated personalized care [59]. Electronic patient-reported outcome measures, telepsychiatry consultations, and digital adherence tracking can provide continuous, real-time monitoring of patients’ psychological state and treatment engagement, enabling proactive adjustments to care plans. [23]. These tools are particularly relevant for high-volume oncology centers and geographically diverse patient populations, where in-person psycho-oncology resources may be limited. In addition to molecular profiling and digital health tools, emerging diagnostic imaging technologies are increasingly contributing to precision breast cancer care.

Advanced imaging modalities, including functional, molecular, and hybrid imaging approaches, may enhance biological risk stratification, early response assessment, and therapy monitoring, complementing genomic and biomarker-driven strategies [60]. By providing dynamic and spatially resolved information on tumor biology, these techniques may further refine personalized treatment planning. Importantly, improved diagnostic accuracy and clearer prognostic information may also indirectly influence psychological outcomes by reducing uncertainty, supporting shared decision-making, and facilitating patient engagement within personalized care pathways.

From a research perspective, further studies are needed to clarify the mechanisms linking psychological factors to oncological outcomes and to identify the most effective intervention strategies across different patient subgroups. Pragmatic clinical trials and real-world evidence studies focusing on integrated care models can provide actionable insights into optimizing both clinical efficacy and patient-centered outcomes. Importantly, future research should prioritize clinically meaningful endpoints, including treatment adherence, persistence, quality of life, and functional status, alongside traditional oncological outcomes.

Ultimately, advancing toward truly personalized breast cancer care requires a cultural shift in oncology practice. Recognizing psychological and psychiatric dimensions as core determinants of treatment success, rather than optional supportive components, is essential to fully realizing the benefits of precision oncology. By embedding integrated, patient-centered approaches into routine care, oncology centers can maximize the benefits of biological innovation while addressing the full spectrum of factors that shape patient outcomes. Ongoing research should further explore the integration of digital health tools, including electronic patient-reported outcomes, remote psychological monitoring, and tele-psycho-oncology interventions, as scalable solutions to support integrated personalized care, particularly in high-volume or resource-limited oncology settings.

## 9. Conclusions

Breast cancer has become a leading example of precision oncology, with treatment strategies increasingly guided by molecular classification and biomarker-driven therapies. These advances have significantly improved survival and disease control; however, their real-world effectiveness remains variably influenced by patient-related psychological and psychiatric factors that are often insufficiently addressed in routine clinical practice. Without accounting for psychosocial determinants, even biologically optimized therapies may fail to achieve their intended impact in clinical settings.

This narrative review highlights that true personalization in breast cancer treatment extends beyond biological precision. Depression, anxiety, psychological distress, coping styles, and psychosocial vulnerability exert a clinically meaningful impact on treatment adherence, tolerability, quality of life, and, indirectly, oncological outcomes. Addressing these dimensions systematically ensures that precision therapies are delivered effectively, improving both patient-reported and objective outcomes. When these dimensions are not systematically assessed and managed, the potential benefits of precision oncology may be partially undermined.

Integrating psycho-oncology into personalized breast cancer care offers a pragmatic and evidence-based solution to this gap. Structured psychological screening, stratified referral pathways, and targeted psychological and psychiatric interventions represent clinically actionable tools that can be embedded within standard oncological workflows. Embedding these interventions ensures that psychological vulnerabilities are treated proactively, preventing disruptions in care and optimizing the effectiveness of precision therapies. Replicable integrated care models demonstrate that collaboration between oncologists and mental health professionals is both feasible and beneficial, enhancing patient-reported outcomes and supporting sustained treatment engagement.

In conclusion, bridging precision oncology and psycho-oncology enables a more comprehensive, person-centered approach to breast cancer management. Psycho-oncological assessment and intervention are not merely supportive measures but essential components for maximizing clinical effectiveness in modern breast cancer care. Future efforts should focus on standardizing integrated care pathways and promoting multidisciplinary models that address both tumor biology and patient-specific psychological determinants of outcome. Despite growing recognition of the clinical relevance of integrating precision oncology and psycho-oncology, several important research gaps remain. Limited evidence is available on the interactions between biological biomarkers and psychological phenotypes, such as whether specific molecular subtypes, inflammatory profiles, or treatment-related biomarkers are systematically associated with distinct patterns of psychological vulnerability, resilience, or coping. Elucidating these relationships may enable more refined, multidimensional risk stratification.

Future research should explore AI-assisted models capable of integrating biological, clinical, and psychological variables to support personalized decision-making. Multimodal algorithms combining molecular data, imaging features, patient-reported outcomes, and psychosocial indicators may enhance the prediction of adherence, tolerability, and real-world treatment effectiveness beyond biological endpoints alone.

While integrated oncology–psycho-oncology models demonstrate consistent benefits on patient-reported outcomes, evidence regarding their impact on hard clinical endpoints, healthcare utilization, and cost-effectiveness remains limited. Pragmatic trials and real-world implementation studies are needed to evaluate whether integrated care frameworks reduce treatment discontinuation, unplanned healthcare use, and overall system costs, particularly in long-term breast cancer management.

Finally, future efforts should focus on standardizing integrated care pathways and defining measurable quality indicators for personalized breast cancer care. Establishing consensus-driven frameworks will be essential to translate conceptual integration into scalable, sustainable, and truly customized clinical practice.

## Figures and Tables

**Table 1:** Biological vs. Psycho-Oncological Dimensions of Personalization in Breast Cancer Care.

Domain	Precision Oncology	Psycho-Oncology
Primary focus	Tumor biology	Patient psychological and psychiatric profile
Key variables	Molecular subtype, biomarkers, genomic signatures	Depression, anxiety, distress, coping style
Assessment tools	IHC, NGS, multigene assays	Distress Thermometer, HADS, clinical interview
Treatment targets	Tumor growth and progression	Emotional well-being, adherence, coping
Therapeutic strategies	Targeted therapy, endocrine therapy, immunotherapy	Psychological interventions, psychopharmacology
Main outcomes	Survival, response rate, disease control	Adherence, quality of life, symptom burden
Limitations when used alone	Biological response ≠ clinical effectiveness	Limited impact without oncological integration
Added value when integrated	Optimized treatment selection	Improved real-world effectiveness

Note: This table compares the core components of precision oncology and psycho-oncology within personalized breast cancer care. Precision oncology focuses on tumor-specific biological characteristics, such as molecular subtypes and actionable biomarkers, to guide targeted treatment selection and improve disease control. Psycho-oncology addresses patient-related psychological and psychiatric dimensions, including depression, anxiety, distress, and coping styles, which significantly influence treatment adherence, tolerability, quality of life, and real-world clinical effectiveness. The integrated consideration of both domains supports a more comprehensive, person-centered approach to breast cancer management. Abbreviations: IHC, immunohistochemistry; NGS, next-generation sequencing; HADS, Hospital Anxiety and Depression Scale.

**Table 2:** A Replicable Integrated Precision–Psycho-Oncology Model: From Screening to Clinical Outcomes.

Step	Clinical Action	Tools/Interventions	Target Population	Expected Outcomes
1. Screening	Routine psychological assessment	Distress Thermometer, HADS	All breast cancer patients	Early identification of vulnerability
2. Stratification	Risk-based classification	Clinical judgment, history	Low vs. high psychological risk	Appropriate resource allocation
3. Intervention	Tailored psycho-oncological care	CBT, supportive therapy, antidepressants	Patients with distress/depression	Reduced symptoms, improved coping
4. Integration	Multidisciplinary coordination	MDT meetings, shared records	Patients on long-term therapies	Improved adherence and persistence
5. Monitoring	Outcome and adherence assessment	PROMs, adherence tracking	High-risk patients	Optimized treatment effectiveness

Note: This table outlines a stepwise and replicable framework for integrating psycho-oncological care into personalized breast cancer treatment pathways. The model progresses from routine psychological screening to risk stratification, targeted psychological and psychiatric interventions, multidisciplinary integration, and outcome monitoring. By aligning psycho-oncological actions with oncological decision-making, this integrated approach aims to improve treatment adherence, symptom tolerability, quality of life, and overall clinical effectiveness across oncology care settings. Abbreviations: HADS, Hospital Anxiety and Depression Scale; CBT, cognitive-behavioral therapy; MDT, multidisciplinary team; PROMs, patient-reported outcome measures.

## Data Availability

No new data were created.
